# A Case of Stiff Person Syndrome: Immunomodulatory Effect of Benzodiazepines: Successful Rituximab and Tizanidine Therapy

**DOI:** 10.1097/MD.0000000000000954

**Published:** 2015-06-12

**Authors:** Przemyslaw Zdziarski

**Affiliations:** From the Department of Clinical Immunology, Lower Silesian Center for Cellular Transplantation, Wroclaw, Poland.

## Abstract

Stiff person syndrome (SPS) is a rare autoimmune disease. Most patients have high-titer antibodies against glutamate decarboxylase (GADAb), which is without practical value in disease monitoring. Benzodiazepines are the first line drugs, but long-term use is not well characterized.

This report demonstrates ineffective benzodiazepine therapy of SPS that prompts tachyphylaxis, loss of responsiveness, and finally benzodiazepine withdrawal syndrome. Convulsion and anxiety correlate with high level of creatine phosphokinase (CK). Although tonus and spasm attacks were successfully controlled by tizanidine, glutamate release inhibitor, the immune response, and autoimmune diabetes development require the plasmapheresis, mycophenolat mofetil, and rituximab therapy that results in a significant decrease of GADAb, impaired glucose tolerance (IGT), lactate dehydrogenase (LDH), and CK normalization. Unfortunately, reintroduction of benzodiazepine was a source of rapid and high increase of CK, LDH, GADAb titer (up to 1:15,000), IGT, and SPS relapse. Contrary to previous publications, we observed IGT that correlated with high anti-GAD level, but without high immunogenetic susceptibility to haplotype human leukocyte antigens-DR3, DQw2.

This preliminary observation and the last finding of immunomodulatory properties of peripheral benzodiazepine receptor suggest that increased antigenic stimulation during benzodiazepine therapy and glutamatergic hyperactivity could account for convulsions observed in SPS. Benzodiazepine withdrawal prompted alternative muscle relaxant therapy (tizanidine). Muscular and brain abnormalities observed in SPS indicate that noncardiac CK level may be a useful tool in SPS therapy monitoring.

## INTRODUCTION

Stiff person syndrome (SPS) is a humoral autoimmune disorder characterized by the impairment of major inhibitory transmitter system mediated by γ-amino butyric acid (GABA). The lack of GABA-dependent signal causes progressive stiffness and rigidity of truncal muscles accompanied by cocontraction of agonist-antagonist muscles.

In physiological circumstances, GABA blocks the stimulatory/excitatory signal by postsynaptic GABA (A)R-derived hyperpolarization. Although antibodies against glutamate decarboxylase (GADAb) are crucial in GABA synthesis blocking, the GADAb level did not correlate with disease severity;^1^ surprizingly little attention has been paid to the clinical disease monitoring. Benzodiazepines are the first line drugs, some researchers use the good response to benzodiazepines as one of diagnostic criteria,^2,3^ but autoimmune response to GAD and outcome of long-term intensive SPS therapy has not been described. Potential side effects of benzodiazepines prompted alternative muscle relaxant therapy. A severe case of nonparaneoplastic (primary autoimmune etiology) SPS with impaired glucose tolerance (IGT) development is presented in this report.

Patient gave written informed consent for this publication.

## CASE PRESENTATION

A 39-year-old woman without underlying malignancy was previously treated with diazepam and remained ambulant.

**FIGURE 1:**
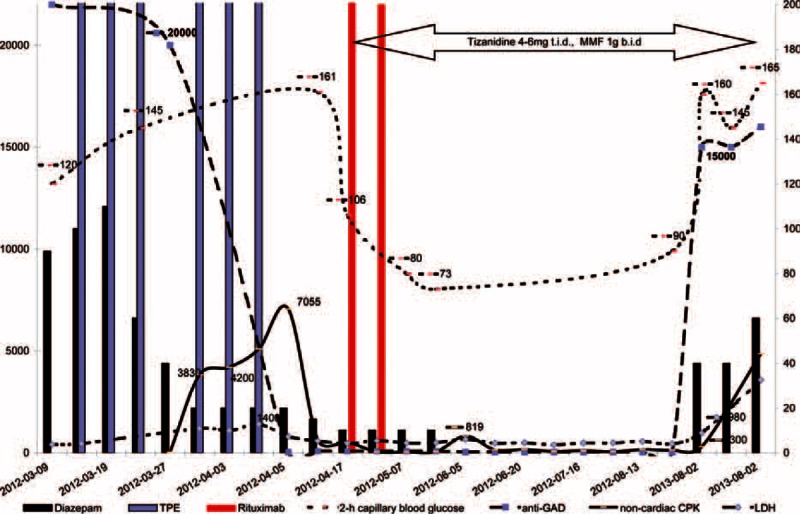
Immunomodulatory effect of benzodiazepines. The effect of diazepam (DZ) withdrawal and plasmapheresis treatment followed by immunosupprression on serum anti-GAD antibody (GADAb) titer, glucose intolerance, and noncardiac creatine kinase (CK) (CK-MM + CK-BB) in SPS patient. Single course of rituximab (375 mg/m^2^ infusions on day 1 and 8) followed by mycophenolate mofetil (1 g b.i.d.) and tizanidine (4–6 mg t.i.d.).

The woman was admitted to an immunological department due to muscle hypertonia with episodic attacks of painful spasms, affecting predominantly axial muscles. Benzodiazepine monotherapy proved to be ineffective despite a high dose of diazepam (50 → 100 mg/24 hours gets out of control). Very high titer GADAbs (>1: 20,000) were observed (Figure 1). Furthermore, an immunogenetic element was tested in individual susceptibility to SPS and in latent autoimmune diabetes of adults (LADA) (Table 1).

**TABLE 1:**
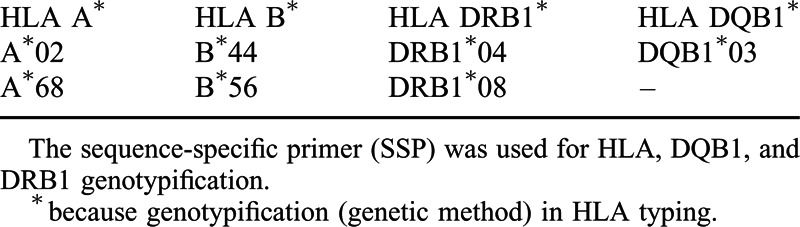
SSP-HLA Typing Result

### Therapeutic Intervention

Following the clinical manifestation and laboratory investigations (the patient satisfying M.C. Dalakas criteria),^3^ the diagnosis of autoimmune SPS was established and the patient was urgently treated with 2 courses of plasmapheresis [therapeutic plasma exchange (TPE)].

Since the IGT is a risk factor for the development of diabetes mellitus, the patient underwent a 75 g oral glucose tolerance test (OGTT) before, during and after plasmapheresis as well as rituximab therapy: the cut-off point was set at blood glucose of 140 mg/dL (7.8 mmol/L) as previously described^4,5^ and in accordance with the WHO criteria.

### Follow-Up and Outcomes

After the second course of TPE common complications—hypoalbuminemia and anemia—were observed as the result of large volume plasmapheresis (total volume > 4000 mL). Biochemical and laboratory investigations showed high muscle involvement: very high level of creatine kinase (CK) (brain CK-BB plus muscle type CK-MM, but not cardiac CK-MB isoenzyme). Unfortunately, the refractory status epilepticus-like symptoms were observed, continuing seizures despite adequate initial pharmacologic treatment and essential GADAb reduction after TPE. Immunosuppressive agents were added a single course of rituximab (375 mg/m^2^ on day 1, 8) followed by mycophenolat mofetil (1 g b.i.d.) (Figure 1). Interestingly, the initial exteroception and spasm were confined to the muscles of the trunk followed by sleeplessness, anxiety, myoclonic jerks and further life-threatening seizures, tachycardia, sweating, and vegetative symptoms after TPE. Benzodiazepine withdrawal syndrome developed because the patient took benzodiazepines for a long time (at increasing dose, out of control), serum drug concentration was drastically reduced by plasmapheresis. Initially we did not notice IGT, but it was visible later, after diazepam withdrawal. We observed successful therapy of autoimmune phenomena: SPS and IGT/LADA—gradually the patient became able to walk, read books (GADAb was below 1:80 titer, rituximab therapy reestablished OGTT) (Figure 1). Further on, after benzodiazepine withdrawal, the tonus and spasm attacks were successfully controlled by tizanidine, and the patient was discharged.

Unfortunately, after the reintroduction of benzodiazepine (out of control), rapid and high increase of GADAb and IGT (defined as 2-hour glucose levels of 140 to 199 mg/dL)—LADA and SPS relapse were observed.

## DISCUSSION

**FIGURE 2:**
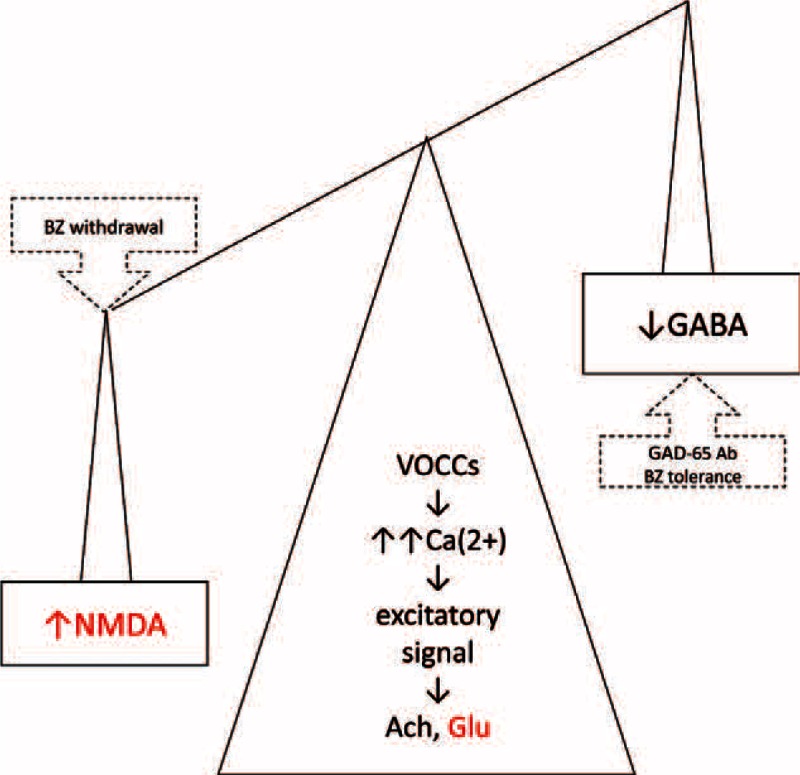
Tizanidine therapy in stiff person syndrome (SPS). The inhibitory and excitatory equilibrium disturbance in SPS and long-term benzodiazepine (BZ) therapy. Red font: points of tizanidine therapy. Anti-GAD65Ab = antibodies against glutamate decarboxylase 65 kDa, GABA = γ-amino butyric acid; Glu-glutamate, NMDA = *N*-methyl-D-aspartate, VOCC = voltage-operated Ca(2+) channels.

Physiologically the competition between major excitatory and inhibitory systems allows for the elastic tension of living muscles that facilitate the response to stimuli. In SPS the major inhibitory system, mediated by GABA, is impaired. Accordingly, the main excitatory transmitter system mediated by glutamate and its receptor excites uncontrollable electric signal, that is, the source of muscle stiffness and sudden onset of spasms. *N*-methyl-D-aspartate (NMDA) receptors engagement activates nitric oxide synthase and nitric oxide release. Furthermore, presynaptic GAD65 function is directly correlated with the glutamate receptor level.^6^ The same neurological abnormalities are the source of benzodiazepine withdrawal symptoms as a result of uncontrolled excitatory signal, prolonged abuse of diazepam leads to GABA-receptor stimulation, potentiation, and then neuroadaptation that prompt the decrease of GABA and increase of NMDA receptor activity (Figure 2). The inhibitory and excitatory equilibrium is disturbed by GADAb, benzodiazepine tolerance and may be restored by tizanidine therapy. It is crucial to achieve the tonus control by indirect NMDA blocker therapy (ie, tizanidine, glutamates release inhibitor) without pharmacodynamic tolerance (reduced responsiveness). We suggest that possible GADAb-induced GABA-receptor blocking and glutamatergic hyperactivity could account for convulsions in SPS. It corresponds with the data saying that tizanidine pretreatment decreased significantly the incidence of convulsion.^7^

SPS is a CNS disorder characterized by increased muscle tone and prominent agoraphobia and anxiety. Moreover, during brain injury total CK may be increased. CK-BB isoenzyme is rarely encountered clinically, but involvement of brain and striated muscle in the manifestation of SPS prompts the use of noncardiac CK isoenzymes (CK-MM and CK-BB together) and lactate dehydrogenase in clinical monitoring (Figure 1). Although the antibody-dependent cytotoxicity (ADCC) is the main patomechanism of GAD65Ab-dependent diabetes mellitus, so far the pathogenetic and prognostic role of GAD65Ab level in SPS has been unknown.^8^ Contrary to ADCC, the CNS autoantibodies to GAD may cause predominant functional impairment of GABA neurons (reversible injury). It has been noted that neurological impairments caused by GAD65-Ab can vary according to epitope specificities.^9^ In the context of epitope selection, the human leukocyte antigens (HLA) typing of patients with SPS and diabetes mellitus is crucial,^10^ but success in the HLA predisposition to SPS has so far proved elusive. There was observed glucose intolerance, defined as 2-hours blood glucose >140 mg/dL (7.8 mmol/L), but not typical of DM-1-susceptibility to haplotype HLA-DR3, DQw2.

On the other hand, GAD65 is a dimeric enzyme responsible for catalyzing reaction, so binding by antibody in any place (probably at an allosteric site) may be the source of catalytic inhibition (competitive or not).^11^ GAD65-Ab titer in SPS is much higher than in DM-1,^12^ implying passive transfer of IgG to the central nervous system.

Benzodiazepines are major substrates of cytochrome P450 (CYP3A4). Human CYPs are primarily membrane-associated proteins located either in the inner membrane of the mitochondria or in the endoplasmic reticulum of cells involved in the biosynthesis of immunomodulatory substances, such as lipids and steroid hormones. Unfortunately, the peripheral benzodiazepine receptor (PBR) is a molecule involved also in cholesterol transport through the mitochondrial membrane. Such anti-inflammatory properties may lower the seizure threshold, the coincidence of seizure threshold in the diazepam withdrawal and pretreatment with the cyclooxygenase inhibitor was observed in an animal model.^13^

New insight into benzodiazepine therapy in SPS is provided by immunomodulatory properties of PBR on the outer mitochondrial membrane that suppresses Tumor necrosis factor-induced Vascular cell adhesion protein 1 and Intercellular Adhesion Molecule 1 expression on endothelium^14^ and prevents Formylmethionyl-leucyl-phenylalanine-induced L-selectin, Platelet endothelial cell adhesion molecule-1 on the neutrophil cell surface.^15^ Contrary to peripheral immunosuppressive action in microglia, PBR is expressed from the earliest stages of activation and appears to exert proinflammatory function.^16^

Despite the limitations of the retrospective character, this case summarizes the experience currently available on immunotherapeutic interventions, outcome of long-term benzodiazepine SPS monotherapy, clinical and laboratory disease monitoring, and adverse reactions.

## CONCLUSIONS

Most patients with SPS respond to benzodiazepines, but the required high doses cause unacceptable benzodiazepine dependence. This direct effect in SPS may be escalated by tachyphylaxis, the decrease in the response, increase of the dose of diazepam to restore the original response, and finally the loss of response to the continued or increased dose. Some withdrawal symptoms are identical to those due to that diazepam was originally prescribed. Diazepam causes downregulation of GABA-receptor numbers, but increases GABA production by high expression of GAD65 and antigenic or PBR-derived stimulation for GAD-specific lymphocytes. Tizanidine, a glutamate release inhibitor, may be the therapeutic option without tachyphylaxis (Figure 2). The measurement of CK different isoforms is useful in stiffness monitoring (Figure 1), but the progress in characterizing the CK-BB in brain damage and SPS is still a challenging problem.
